# Bariatric Surgery and Vitamin D: Trends in Older Women and Association with Clinical Features and *VDR* Gene Polymorphisms

**DOI:** 10.3390/nu15040799

**Published:** 2023-02-04

**Authors:** Linconl Agudo Oliveira Benito, Evelyn Mikaela Kogawa, Calliandra Maria de Souza Silva, Fabíola Ferreira Melo, Silvia Helena de Carvalho Sales-Peres, Izabel Cristina Rodrigues da Silva, Margô Gomes de Oliveira Karnikowski

**Affiliations:** 1 Graduate Program in Health Sciences and Technologies, Faculty of Ceilandia, University of Brasília, Federal District, Brasília 72220-275, DF, Brazil; 2 Bauru School of Dentistry, University of São Paulo (USP), Bauru 7012-901, SP, Brazil; 3 Department of Dentistry, School of Health Sciences, University of Brasília, Brasília 70.910-900, DF, Brazil

**Keywords:** Roux-en-Y gastric bypass bariatric surgery, vitamin D, vitamin D receptor, *Fok*I (rs2228570), *Taq*I (rs731236)

## Abstract

(1) Background: Obesity and its comorbidities can cause burdens and limitations. Bariatric surgery (BS) is indicated as a safe procedure to reduce body mass and improve present comorbidities. However, several complications were reported, such as vitamin D [25(OH)D] deficiency. We evaluated if 25(OH)D serum levels relate to clinical characteristics, symptoms, or habits in women after their BS, and whether the vitamin D receptor (*VDR*) gene’s *Taq*I and *Fok*I polymorphisms affected 25(OH)D levels and the total body bone mineral density (TBBMD). (2) Methods: This cohort cross-sectional comparative analytical prospective study consisted of 27 women, 61.6 ± 5.0 years, submitted to BS one year prior at a public reference hospital, DF-Brazil. All participants were asked to follow the physical and dietary activity recommendations and received vitamin D3 supplements. Their anthropometric, biochemical, and immunological measurements and blood samples were obtained. (3) Results: 73.3% of participants had low 25(OH)D levels, and their levels correlated positively with TBBMD and negatively with systolic pressure. *VDR Taq*I did not affect 25(OH)D levels, whereas *VDR FokI*’s allele f presence correlated to a median rise in 25(OH)D levels. Neither polymorphism correlated to TBBMD. (4) Conclusions: 25(OH)D levels were positively correlated with TBBMD, negatively with systolic blood pressure, and were higher in those with the *VDR Fok*I allele *f*.

## 1. Introduction

The quantitative increase in the population’s older adult ratio worldwide is strongly known internationally [1,2]. Some demographic projections argue that by the year 2050, approximately 21% of the world’s population will consist of people in their sixties or older (>60 years) [1,2] and that approximately 80% of these will be living in low- and middle-income classified nations [3]. This increase, however, in life expectancy has not necessarily been in the healthy work-capable older adults’ portion, which could justify an increase in the state pension age, but mainly in the illness-prone, chronically ill portion. Even though some major risk factors have declined with age (e.g., smoking), others have increased—particularly chronic non-communicable diseases (CNCD), such as obesity. In older adults, obesity and morbid obesity are dangerous organic conditions [4,5,6] identified in both industrialized and post-industrialized nations [7]. The increase in obesity in the past 40 years has been staggering, representing approximately two billion people worldwide [2], and might configure the most significant global risk public health has ever met [7,8,9].

For some researchers, obesity is characterized as a systemic inflammatory disorder [10], complex and multifactorial [8,10], highly prevalent [7,8,10], and correlated with several chronic and cardiovascular diseases [8,9,10,11]. Physical inactivity [11,12], adipose tissue accumulation [11,12,13], consequences related to dietary factors [13,14,15], psychosocial aspects [13,14,15], genetic susceptibility [16,17], and obesogenic coefficients [18] are some of the phenomena related to obesity that negatively impact both health [13,14,15,19] and quality of life [14,16].

For other researchers, obesity and its comorbidities can cause complex commitments and limitations in older adults [12,15] regarding their life and existence, for example, in work performance [8,12,18], financial and economic activities, and participatory citizenship in society [12,18]. Among the various diseases and comorbidities related to obesity, we can mention arterial hypertension [11,20], coronary artery disease [20], diabetes mellitus [11,20], dyslipidemia [8,11], obstructive sleep apnea syndrome [8,11], steatohepatitis [18], various types of cancer [11,20], and the deterioration of lower limb torque [21], in addition to an increased risk of death on account of the comorbidities mentioned above [20,22,23].

This way, bariatric surgery (BS) is indicated as a safety procedure [22,24,25] to combat obesity that significantly reduces body mass and improves present comorbidities [22,23,24,25,26,27]. Nonetheless, there are still controversies about its effectiveness in older adults [26,27] and their postoperative recovery [26]. According to some researchers, older patients are less frequently submitted to BS compared to patients in a lower age group [23,26], although their numbers have increased in the last decades to approximately 5–6% of the total BS [23].

In contrast, several BS-related complications have been documented, such as malnutrition, chronic nausea and vomiting, acid reflux, the inability to eat certain foods, and esophageal dilation [11,12]. Among these, malnourishment significantly threatens the health of patients subjected to BS [13,19], as even purely restrictive surgeries can render an approximate 20–50% insufficiency of micronutrients [19]. The gap in knowledge on the potential health problems linked to each type of bariatric procedure must be considered in both pre-surgery and post-surgery stages to manage patients’ long-term weight loss and health. For instance, all types of bariatric operations reported vitamin D [25-hydroxyvitamin D-25(OH)D, with D representing either D2 or D3] deficiency [15], which could result in secondary hyperparathyroidism (SHPT), especially in patients subjected to malabsorptive surgical interventions [14].

Hence, human BS studies should also assess the presence of genetic polymorphisms in vitamin D nutritional metabolic processes/pathways, such as vitamin D’s receptor (VDR) and its metabolizing enzymes. This receptor’s gene, located on chromosome 12 (12q13.11), is expressed in most of the immune system’s cells, including CD4+ and CD8+ T lymphocytes, as well as antigen-presenting cells, such as macrophages and dendritic cells [28]. VDR belongs to the nuclear receptor superfamily of the transcription regulatory factors for steroid hormones, retinoic acid, thyroid hormone, and vitamin D and consists of eleven exons [29]. The VDR protein is encoded by exons II to IX, with exons VII to IX involved in the binding of VDR to its ligand, vitamin D [30]. After 1,25(OH)2D [1,25-dihydroxy vitamin D] binds with the VDR, the receptor interacts with the retinoic acid receptor (retinoid X receptor), forming a heterodimeric complex (RXR-VDR) which, in turn, binds to specific DNA sequences, known as the vitamin D responsive element (VDRE) [31]. The main target organs for 1,25(OH)2D are the intestines, bone, parathyroid glands, and kidneys. However, several other tissues also have VDR [32].

Genetic variations in the VDR gene that change the receptors’ protein conformation could explain significant defects in gene activation and target genes’ transactivation, thus affecting calcium metabolism, cell proliferation, and immune function [33,34]. Interestingly, despite the increasing number of studies scrutinizing genetic variations’ influence on several diseases, only a few have investigated *VDR* polymorphisms [35]. The *VDR* gene has several different genetic polymorphisms, including the single nucleotide polymorphisms (SNPs): *Apa*I (rs7975232, intron 8, +64978 C > A), *Bsm*I (rs1544410, intron 8, +63980 G > A), *Fok*I (rs2228570, formerly rs10735810, exon 2, +30920 C > T), and *TaqI* (rs731236, exon 9, +65058 T > C) [34,35]—all reported to be associated with several diseases [33,35].

The *Taq*I (rs731236) SNP ushers a synonymous change (T > C), as both resultant codons encode the amino acid isoleucine [36]. Although generating a silent mutation, this transition changes some functional characteristics of the protein, with the T allele associated with higher transcriptional activity, mRNA stability, and vitamin D serum level [36,37]. Another functional SNP is the *Fok*I (rs2228570) that modifies the translation initiation codon (C > T) and produces a peptide shorter by three amino acids (424 < 427) with a higher transcriptional activity compared to the original, leading to a change in the VDR protein activity [38,39]. However, this effect appears to be gene-specific and cell-type-specific.

In this context, this study evaluated if 25(OH)D serum levels relate to anthropometric, biochemical, and immunological characteristics and other remarkable characteristics in women twelve months after their bariatric surgery. Furthermore, this study assessed whether there is a difference between 25(OH)D serum level and the total body bone mineral density according to the presence of the *VDR* gene’s *Taq*I (rs731236) and *Fok*I (rs2228570) polymorphisms.

## 2. Materials and Methods

### 2.1. Study Design and Research Participants

This research is a cohort, cross-sectional, comparative, analytical, prospective study with a quantitative and qualitative approach. The research participant sample consisted of 27 older female adults, aged fifty (50) years or more (61.6 ± 5.0 years), submitted to Roux-en-Y gastric bypass bariatric surgery (BS) one (1) year prior at a reference public hospital of the Federal District’s Secretary of State of Health (SES-DF, Brazil), and able to understand, verbalize, and answer the proposed questions (inclusion criteria). Participants were excluded from the study if they had a mental illness, were under the age of fifty (50), did not undergo BS, had their BS performed procedure in less than one (1) year, if BS was not performed with the Hospital Regional da Asa Norte (HRAN), or if they not fit the inclusion criteria established by this research. This sample size (*n* = 27) was calculated by Raosoft software online, keeping a 95% confidence level, an 8% margin of error, population size (number of patients eligible for the inclusion criteria in 2018, *n* = 28), and a 50% response rate, totaling *n* = 24; but as we considered a 10% loss rate, the final sample size of 27. The Federal District State Department of Health (SES-DF)’s Health Sciences Teaching and Research Foundation (FEPECS)’s Research Ethics Committee (CEP), under opinion number 1.910.166, approved this study. All participants signed the informed consent form (ICF).

### 2.2. Clinical and Laboratory Evaluation

For their clinical and laboratory evaluation, all the participants were asked to follow the physical and dietary activity recommendations detailed in the clinical protocols for identifying, assessing, and treating obesity and being overweight in adults [21] and received vitamin D3 supplements (1000 IU/day). The age, height, weight, and body mass index (BMI) were obtained from the nursing consultation’s medical record and responses to the collection instruments.

The clinical data and the blood samples were collected at 12 months post-surgery. All the biochemical parameters, i.e., triglycerides (TG), fasting blood sugar (FBS), and minerals, were assessed in the same laboratory using standard commercial methodologies. The total vitamin D [25-hydroxyvitamin D2 and D3, 25(OH)D2 and D3] serum levels were also measured using a standard commercial chemiluminescence kit—the LIAISON 25(OH)D Total Assay (DiaSorin, Saluggia, Italy) with a functional sensitivity <10 nmol/L, 100% specificity, the dynamic range between 4.0–150 ng/mL, and the coefficient of variation within assay of 2.3 and inter-assay of 7.80.

TNF-α, IL-6, IL-10, and IL-2 serum levels were measured using an enzyme-linked immunosorbent assay (ELISA) technique—Human ELISA Kit (Invitrogen, San Diego, CA, USA; Thermo Fisher Scientific, Schwerte, Germany)—these assays detect only human cytokines. The minimum detectable concentrations considered in our laboratory were 4.8 pg/mL for TNF-α, 1.1 pg/mL for IL-6, 2.0 pg/mL for IL-10, and 1.0 pg/mL for IL-2.

### 2.3. Genotype Analysis

For genotyping, deoxyribonucleic acid (DNA) was extracted from the participants’ collected blood using Invitrogen’s PureLink^®^ Genomic DNA Mini (catalog #K1820-02, lot #19339891; Invitrogen, Waltham, CA, USA), with a 20 ng/μL average concentration.

*VDR Taq*I (rs731236, exon 9, +65058 T > C) polymorphism was genotyped using the polymerase chain reaction combined with the restriction fragment length polymorphism (PCR-RFLP) based analysis. The primers used were forward/sense 5′-CAG AGC ATG GAC AGG GAG CAA G-3′ and reverse/antisense 5′-GCA ACT CCT CAT GGG CTG AGG TCT CA-3′ [40]. The DNA’s amplification was performed using the following thermocycling conditions: 95 °C for 5 min (initial denaturation), followed by 35 cycles of denaturation at 94 °C for 30 s, annealing at 65 °C for 30 s, and extension at 72 °C for 30 s. The final extension occurred at 72 °C for 10 min. The PCR product is a 740 bp fragment amplified from the *VDR* gene’s exon 9 region. After its 3 h digestion with the *Taq*I restriction enzyme (cat#EN-142S, Jena Bioscience, Jena, Germany), the polymorphism is cleaved into three bands of 290, 245, and 205 bp, defined as mutant *t* (C) allele, while the appearance of two 490 and 245 bp fragments indicates the presence of the ancestral allele *T* (T). Therefore, the TT (TT) genotype is defined by 490 and 245 bp presence, the *Tt* (TC) genotype by 490, 290, 245, and 205 bp, and the tt (CC) genotype by 290, 245, and 205 bp.

*VDR Fok*I (rs2228570, formerly rs10735810, exon 2, +30920 C > T) SNP analysis was also performed by PCR-RFLP. The primers’ sequences were as follows: forward/sense 5′-AGCTGGCCCTGGCACTGACTCTGCTCT-3′ and reverse/antisense 5′-ATGGAAACACCTTGCTTCTTCTCCCTC-3′ [41]; with thermocycling parameters as follows: 95 °C for 5 min (initial denaturation), followed by 35 cycles of denaturation at 94 °C for 30 s, annealing at 61 °C for 30 s, and extension at 72 °C for 30 s, and one final extension cycle at 72 °C for 5 min. The 280 bp PCR product was then digested with the *Fok*I restriction enzyme (cat# FD2144, Thermo Scientific CA, USA) and incubated at 37 °C for 15 min. The digested products were 265 bp for the C (*F*) allele and 169 and 96 bp for the T (*f*) allele. Whereas the *FF* (CC) genotype is defined by 265 bp presence, the *Ff* (CT) genotype is defined by 265, 169, and 96 bp, and the *ff* (TT) genotype is defined by 169 and 96 bp.

The *VDR Taq*I (rs721236) and VDR *Fok*I (rs2228570) polymorphism genotypic patterns were determined by running the digested products on a 3% agarose gel.

### 2.4. Body Composition Assessment

Each set (two repetitions per participant) of individual’s dual-energy X-ray absorptiometry (DXA) measurements were performed on the same day by the same operator, with the participants under at least 4 h of fasting and 24 h of exercise abstinence to ensure adequate hydration conditions.

Lunar Prodigy Advance equipment (General Electric Systems, Madison, WI, USA) was employed to determine fat mass (FM, g), lean mass (LM, g), and total body bone mineral density (TBBMD, g). The DXA device was calibrated with phantoms before each set of measurements. Our laboratory’s variability coefficient was 1.03, 1.35, and 0.83% for FM, LM, and TBBMD, respectively.

### 2.5. Statistical Analysis

For statistical analysis, absolute and relative frequency distribution was applied for categorical variables and quartiles for continuous variables—with continuous data expressed as mean ± standard error (SE) or percentiles. Spearman’s coefficient was used to test the correlation between the continuous data of anthropometric, biochemical, and immunological measures and 25(OH)D levels. For the clinical characteristics expressed as categorical data or genotypic frequency, the evaluation of the difference in 25(OH)D serum levels/total body bone mineral density (TBBMD, g) ratio between the groups was evaluated by non-parametric Mann-Whitney U test or Kruskal-Wallis test, because the assumptions of normality were not observed. The chi-square test, with one degree of freedom, analyzed the Hardy-Weinberg equilibrium adherence to the genotypic frequency in controls. The tests were performed with SPSS software version 28.0 (SPSS Inc., Chicago, IL, USA), adopting a significance level of 5.0%.

## 3. Results

### 3.1. Vitamin D Association with Anthropometric and Biochemical Measurements

Vitamin D [25-hydroxyvitamin D, 25(OH)D] serum levels in women after 12 months of bariatric surgery averaged 27.31 ± 7.71 ng/mL. In this group, 11 women (40.0%) had dangerously low levels, up to 25.0 ng/mL, while 20 women (73.3%) had up to 30 ng/mL (standard range in the literature). The minimum value found was 12.50 ng/mL, and the maximum was 40.70 ng/mL.

25(OH)D’s possible associations with anthropometric, immunology or biochemical parameters were assessed by calculating the association’s Spearman correlation coefficients. Table 1 displays 25(OH)D correlations with the selected variables. 25(OH)D serum levels significantly correlated with total body bone mineral density (TBBMD, g) (ρ = 0.514 *, P = 0.010) and were negatively associated with systolic pressure (ρ = −0.711 *, P = 0.049) at 12 months (r = −0.219, P = 0.041) after surgery.

On the other hand, 25(OH)D serum levels correlations with diastolic pressure (mmHg), body mass index (BMI, kg/m^−2^), magnesium, vitamin B12, TSH, T3, T4 total, insulin, fasting blood glucose, total cholesterol, triglycerides, HDL, LDL, VLDL, non-HDL cholesterol, total lipids, uric acid, sodium, potassium, chlorine, calcium, IL-2 (pg/mL), TNF-A (pg/mL), IL-6 (pg/mL), IL-10 (pg/mL), fat mass (FM, g), and lean body mass (LBM, g) were not significant (Table 2).

### 3.2. Participants’ Vitamin D Serum Levels and Other Clinical Signs and Symptoms

The participants were also evaluated regarding the median difference in 25(OH)D serum levels and the presence/absence of other clinical characteristics, symptoms, or habits, such as smoking, alcoholism, hypertension, depression/anxiety, fibromyalgia, neuropathy, arthralgia or myalgia, frequent thirst, and getting up at night to drink water, the loss or alteration of taste, xerostomia (dry mouth), photophobia, itching or rash (pruritus), tingling or numbness, pain in the lower limbs, decreased sweating (perspiration), and altered sexual performance. None of these characteristics were related to changes in the 25(OH)D serum levels (Table 2).

### 3.3. Vitamin D Receptor (VDR) Gene Polymorphisms and Their Relationship with Vitamin D [25(OH)D] Serum Levels and Total Body Bone Mineral Density

After determining that the *VDR Taq*I and *VDR Fok*I polymorphisms’ genotypic distribution obeyed the Hardy–Weinberg equilibrium (P > 0.05), we ultimately verified whether the participants’ vitamin D receptor genetic polymorphism altered their 25(OH)D serum levels. For VDR *Fok*I polymorphism, the mutant allele f presence correlated to a median rise in 25(OH)D serum level both in the genotypic distribution (P = 0.005) and in the dominant model (*FF* versus *Ff + ff*, P = 0.001) evaluation. In comparison, no differences were found regarding the *VDR Taq*I polymorphism. Furthermore, neither of their presences was related to total body bone mineral density (TBBMD). All these analyses are presented in Table 3.

## 4. Discussion

Many nutrients, co-dependent or not, are influenced simultaneously by genetic and hormonal factors, reciprocal interaction with various lifestyle modifiers, or a combination of these. Due to the complexity of these interactions and the biological factors’ dominant influence, nutrients’ effects might be masked and hard to distinguish. Notably, some arguments about the potential impact of micronutrients (e.g., minerals and vitamins) on body health (e.g., bones, muscles, among others) are either just theoretical presumptions, either untested or unproven in human studies or based on animal studies. All this complexity might explain the controversial or inconsistent findings regarding the contribution of a single or a group of nutrients to body health encountered in many studies [42].

The present study found that 73.3% of patients who underwent Roux-en-Y gastric bypass a year prior, even when using vitamin D3 supplements (1000 IU/day), had low vitamin D [25(OH)D] serum levels (up to 30 ng/mL) and that their 25(OH)D serum levels positively correlated with their total body bone mineral density (TBBMD, g).

Vitamin D deficiency, considered one of the main determinants of senile osteoporosis, is much more frequent than imagined in the older adult population, making minimalizing these neuromuscular effects relevant in preventing osteoporotic fracture. Levels of 25(OH)D serum that are lower than 80 nmol/L (approx. <32 ng/mL; 1 ng/mL = 2.5 nmol/L) [43], vitamin D’s current functional status indicator, are associated with reduced calcium absorption, osteoporosis, and an increased fracture risk, with the classic histological alterations of osteomalacia and rickets already evident, with the deficient mineralization of the osteoid matrix [44,45,46]. In this situation, hypocalcemia and hypophosphatemia may be manifest [47].

The high vitamin D deficiency prevalence in older adults could have several causes. For instance, after sun exposure, the synthesis of cholecalciferol (vitamin D3) in the skin is less effective in old age due to a decline in cutaneous 7-dehydrocholesterol levels, roughly 25% lesser in a 70-year-old than in young people [48,49,50]. This reduction worsens by decreased exposure to sunlight due to the immobility, lack of transport, and social isolation usually associated with aging. Another contributing factor is the increase in body fat with aging, which leads to a larger distribution volume for the fat-soluble 25(OH)D3, decreasing the 25(OH)D3 bioavailability [51].

Regarding vitamin D3 supplementation, older adults typically need a supplemental oral intake of approximately 1300 IU/d to reach the lower end of the 25(OH)D optimal range. Multiple vitamin D and its metabolites preparations are commercially available for supplement use—the two most common are ergocalciferol (vitamin D2) and cholecalciferol (vitamin D3), and both formulations are absorbed similarly [52]. However, when used in a large single dose (50,000 IU), vitamin D3 formulation maintains 25(OH)D serum levels more efficiently than vitamin D2, given that 25(OH)D blood levels last three days before dropping rapidly after a vitamin D2 dose compared to two weeks before gradually declining after vitamin D3 dose [53,54]. On the other hand, when considering a 1000 IU daily dose, vitamin D2 was as effective as vitamin D3 in sustaining 25(OH)D serum levels [53,54].

Regarding older patients, there are a few studies involving older people who underwent bariatric surgery, so we analyzed studies with adults and older adults. According to some researchers, patients undergoing BS may be more likely to develop some nutritional deficiencies, such as vitamin D (25(OH)D) deficiency [55,56]. A study by Santos et al. (2020) [55] [646 obese patients submitted to BS, mean age 41.3 ± 10.8, and 75% female] found that 79.1% had insufficiency/deficiency of vitamin D in the preoperative and the 6-month postoperative periods; however, patients submitted to the gastric sleeve (GS) technique showed better vitamin D levels than those undergoing Roux-en-Y gastric bypass (RYGB). A study by Nascimento (2022) [56] [156 patients that underwent GS BS, mean age 40 ± 11.17, and 78.4% female] confirmed this difference in postoperative vitamin D levels between the two methods, as the GS technique associated with better vitamin D levels than RYGB, even though the RYGB technique improved the lipid profile more effectively. In this sense, the hypovitaminosis D incidence is high, both pre and postoperatively, for SG BS as it is for RYGB BS [55,56].

Analyzing BS performance, Sebastião (2019) [57] analyzed 57 people aged 18 years or more, who had undergone RYGB BS at least five years prior. They pointed out that its execution can bring some adverse effects, mainly related to bone and muscle metabolism, such as, for example, the deficit in the absorption of calcium and vitamin D by the intestine [57]. Likewise, Biagioni (2011) [58] study analyzed 25 female (mean age 40.3 ± 8.96) subjects submitted to RYGB BS and verified a reduction in the absorption and ingestion of nutrients essential for the body homeostasis, in particular, bone homeostasis [58].

The issue of vitamin D levels in people undergoing BS constitutes a fundamentally important topic, with some researchers defending that low vitamin D serum levels are directly associated with obesity and prescribing its supplementation after this surgical procedure to help control body weight [59]. Others believe that, at least regarding older patients, several factors might also be related to vitamin D levels. Machado (2016) [59] analyzed vitamin D serum levels in 75 patients submitted to RYGB and SG BS methods and determined that, after the procedures, the vitamin D levels increased and the vitamin D deficiency frequency decreased.

A Vivan et al. (2019) [60] study analyzed vitamin D depletion and its associated factors in 291 patients, with a mean age of 44.9 (SD 10.7), undergoing BS in southern Brazil and identified a high vitamin D insufficiency/deficiency prevalence. More than half of these patients (55.3%) had a vitamin D deficiency (serum 25(OH)D ≤ 19.9 ng/mL), and 37.1% had insufficient levels (20–29.9 ng/mL), requiring vitamin D supplementation in patients aged over 60 years. The vitamin D deficiency was more prevalent in patients with higher BMIs [PR 1.02; 95% CI (1.00–1.03)], with the highest fasting blood glucose [PR 1.01; 95% CI (1.00–1.01)], and in non-white patients [60].

In patients diagnosed with class II or class III obesity and referred for BS, vitamin D deficiency was verified, according to studies conducted in Brazil, Lebanon, and Singapore [60,61,62]. Ong et al. (2018) [61] evaluated obese people submitted to BS in Singapore and perceived that vitamin D deficiency is prevalent, regardless of ethnicity; in particular, in the elderly, women, and those with a higher waist circumference and body fat percentage, being significantly associated with a lower vitamin D serum level.

It is not by chance that some researchers point out the lack of an international consensus on the ideal vitamin D supplementation scheme, be it its dosage or frequency, for patients before and after BS [59,63].

Interestingly, a systematic review by Compher et al. (2008) [64] analyzed the connection between obesity, vitamin D, and obesity surgery’s impact on vitamin D status, and described that the mean 25(OH)D serum level was low (<80 nmol/L) in most patients preoperatively and remained unrestored postoperatively. Notably, secondary hyperparathyroidism and bone loss were likewise typical in these patients, especially when the obesity surgery included a malabsorptive component. This systematic review also noted that the periodic postsurgical vitamin D supplementation has been unsatisfactory in overcoming secondary hyperparathyroidism or reestablishing the optimal vitamin D range. This gap in understanding the mechanisms behind vitamin D deficiency in severe obesity complicates establishing well-defined evidence-based corrective actions. 

Moreover, our study found that after 12 months of bariatric surgery, 25(OH)D serum levels correlated negatively with systolic pressure (ρ = −0.711 *, P = 0.049). However, the median 25(OH)D level difference between patients with hypertension reports and those without was insignificant, despite the high frequency of hypertensive patients in the sample (85.2%). A possible limitation might be that participants’ blood pressure and 25(OH)D levels were measured only at a single time point.

This observation of high blood pressure association to 25(OH)D serum levels started the research into vitamin D involvement in cardiovascular disease pathogenesis [65,66]. Vitamin D may protect against hypertension development as seen by the seasonal variation in blood pressure—lower values in the summer and higher in the winter (an inverse correlation with UV light exposure and circulating 25(OH)D levels) [67]. Similarly, a Judd et al. [68] study with mostly non-hypertensive participants reported a reduction in the age-related increase in systolic blood pressure in patients with adequate 25(OH)D levels (>80 nmol/L), i.e., 25(OH)D-sufficient participants have lower systolic blood pressure (0.40 mmHg/year) compared to deficient and insufficient ones. Wang et al. [69] also reported that participants with low 25(OH)D serum levels had a higher risk of developing cardiovascular events, including incident hypertension, than 25(OH)D sufficient ones. This inverse correlation was maintained even after adjusting for age, sex, ethnicity, and physical activity [70], although further adjusting for body mass index (BMI) and PTH reduced this effect, implying that PTH might mediate most of the 25(OH)D and blood pressure association [71].

However, Snijder et al. [72] observed no positive effects of vitamin D supplementation on blood pressure in the general older adult population but noted that PTH might be a potentially modifiable determinant. On the other hand, in a cohort study by Rei et al. [73] with over 1000 participants (>40 years), neither PTH in women nor 25(OH)D levels in either sex was related to metabolic syndrome, including hypertension.

Unfortunately, observational studies cannot prove causality. Therefore, to test for causality, many intervention studies investigated how dietary vitamin D supplementation affects hypertension. Short-term high vitamin D3 (4000 IU) doses combined with calcium supplements also lowered blood pressure in older German women more than calcium alone [74]. However, Forman et al. [75] reported significantly higher rates of lower circulating 25(OH)D levels and hypertension in Blacks than in Whites—each 1 ng/mL of vitamin D3 (cholecalciferol) supplementation increased in plasma 25(OH)D significantly reduced 0.2 mmHg in systolic pressure but had no effect on diastolic pressure. In contrast, Kunutsor et al. [76] performed a pooled random effects meta-analysis of weighted mean differences across 16 vitamin D supplementation trials and found a non-significant reduction in both systolic and diastolic blood pressure. Interestingly, the diastolic blood pressure was significantly reduced in participants with pre-existing cardiometabolic disease.

Our study found no difference in 25(OH)D serum levels of the participants diagnosed with anxiety/depression (40.7%; N = 11) compared to those without anxiety or depression.

Although growing evidence points to a vitamin D role in depression’s pathobiology and treatment, this evidence is inconsistent in many aspects needing more randomized controlled trials to determine whether this association is causal. Menon et al.’s [77] narrative review found that vitamin D levels’ inverse correlation to clinical depression might be driven by vitamin D’s homeostatic, trophic, and immunomodulatory effects, though this association’s directionality remains unclear. Furthermore, a systematic review and meta-analysis of randomized controlled trials (all published before January 2019) reported in ten studies (total participants = 3336; median duration = 12 months) that high vitamin D supplementation (≥4000 IU) but not lower vitamin D supplementation levels (<4000 IU) correlated with reduced depressive symptoms. Differences in baseline 25(OH)D serum levels before supplementation and the depression assessment scales did not affect this association, grading the overall quality of evidence as ‘moderate’ [78]. In contrast, Penckofer et al. [79] double-blind, randomized, active comparator-controlled trial reported no difference in the dosing effect of vitamin D3 supplementation in treating depressive symptoms in women with significant depressive symptoms, type 2 diabetes (T2DM), and low 25(OH)D levels that received weekly oral or vitamin D3 supplementation (50,000 IU) or an active comparator (5000 IU) for six months.

In our study, 40.7% (N = 11) of the women reported frequent thirst, 29.6% (N = 8) difficulty chewing dry foods, 48.2% (N = 13) xerostomia (dry mouth), and 48.2% (N = 13) reported getting up at night to drink water, but these reports were also unrelated to 25(OH)D serum levels.

Nonetheless, Kong et al.’s experimental study [80] investigated vitamin D’s association with water and electrolyte homeostasis. Vitamin D receptor (VDR)-null mice had polyuria with normal urine osmolarity due to high salt excretion. This polyuria is not attributable to impaired renal fluid handling (similar to the wild-type’s urinary responses to water restriction and vasopressin) or increased intestinal salt absorption (maintained increased water intake and urinary output despite a salt-deficient diet) but rather by the increase in systemic and brain angiotensin II (dramatically upregulated in the VDR-null mice’s kidney and brain compared to wild-type)-induced increase in water intake. On the other hand, researchers identified that 1,25 dihydroxy vitamin D3 downregulates renin expression; thus, vitamin D deficiency or defects in the VDR signaling might lead to renin overexpression and renin-angiotensin system (RAS) activation that might cause renal and cardiovascular injuries and other detrimental effects (RAS activation in other tissues) [81]. Rephrasing vitamin D may play a physiological role in maintaining the renal and cardiovascular systems’ homeostasis via suppressing the RAS.

Regarding *VDR Fok*I and *VDR Taq*I polymorphisms, we found that *VDR Fok*I polymorphism’s mutant allele *f* presence correlated to a median rise in 25(OH)D serum level, both in the genotypic distribution (P = 0.005) and in the dominant model (*FF* versus *Ff + ff*, P = 0.001) evaluation. In contrast, *VDR Taq*I polymorphism did not affect 25(OH)D serum levels. Neither polymorphism correlated to total body bone mineral density (TBBMD).

Studying genetic backgrounds is vital in understanding the context of obesity [82,83]. For instance, the *VDR* gene is highly polymorphic and has many SNPs that might affect its functionality by altering its gene expression, mRNA stability, protein translation efficiency, and protein sequence [84]. These changes might alter the VDR binding pattern with vitamin D or its analogs, thus, changing its related signaling pathways. VDR expression and nuclear activation are necessary for vitamin D’s effects. In this respect, many epidemiological studies have compared case and control groups to test possible linkages between *VDR* polymorphisms and several diseases, including its role in bone biology, renal diseases, diabetes, and other conditions, such as obesity [85]. To illustrate, in fat cells (adipocytes), vitamin D or its analog binds to VDR proteins and acts as a regulator agent in adipocytes’ differentiation and metabolism, and, consequently, alterations in this binding might influence the context of obesity [86].

In the context of obesity/metabolic syndrome/diabetes, some studies have evaluated the 25(OH)D serum level relationship with *VDR* gene polymorphisms. An inquiry by Zaki et al. (2017) [87] included 201 obese Egyptian women with vitamin D deficiency and 249 obese age-matched healthy controls with adequate 25(OH) levels (ages: 25–30). Women with *VDR* mutant alleles for *Apa*I (*Aa + aa*), *Fok*I (*Ff + ff*), and *Taq*I (*Tt + tt*) had significantly lower 25(OH)D serum levels and higher HOMA-IR and blood pressure than those with *VDR* wild-type genotypes: *Apa*I (AA), *Fok*I (*FF*), and *Taq*I (*TT*), respectively [87]. Another study (cross-sectional with 277 patients) assessed the associations between vitamin D deficiency, *VDR* gene polymorphisms (*Apa*I, *Bsm*I, *Fok*I, and *Taq*I), and cardiovascular risk factors in T2DM Caribbean patients. They reported that the vitamin D deficiency rate was higher in T2DM patients and correlated with the *Apa*I and *Fok*I polymorphisms and cardiovascular risk profile. Therefore, *VDR* polymorphisms might explain why vitamin D deficiency is more frequently present in some T2DM patients [88]. In another cross-sectional study with 697 middle-aged Russian women, *Apa*I and *Bsm*I polymorphisms and vitamin D deficiency correlated with metabolic syndrome parameters [89].

Other research examines the same *VDR* polymorphisms and 25(OH)D serum levels in a population but aims to understand distinct illness processes. A survey conducted by Hossein-Nezhad et al. (2014) determined that vitamin D deficiency correlated with the *VDR Fok*I polymorphism in 760 Iranian patients who had undergone angiography due to suspected coronary artery disease (CAD), as vitamin D deficiency that is more prevalent in CAD patients might be result from *Fok*I polymorphism [90]. At the same time, Rashedi et al. (2014)’s study observed that increases in 25(OH)D serum levels in individuals with *VDR Fok*I’s *ff* genotype and low 25(OH)D serum levels might protect them against active tuberculosis [91].

Our study involves older Brazilian women living in Brazil’s central-western region. In Brazil’s southern region, investigators determined the vitamin D deficiency prevalence in girls and found a high vitamin D deficiency/insufficiency prevalence in their sample. They also investigated the vitamin D levels’ association with the *VDR* gene’s *BsmI*, *ApaI*, and *TaqI* polymorphisms and their haplotypes’ distribution and their wild genotypes, and the GGT (*BAT*) haplotype correlated with lower 25(OH)D serum levels [92]. A cohort study by Pereira-Santos et al. (2019) [93], with 270 pregnant women living in northeastern Brazil, evaluated the associations between *VDR* gene polymorphisms, maternal 25(OH)D concentration, and gestational outcomes. They found that participants with the *VDR Taq*I’s *tt* genotype had a higher 25(OH)D concentration during gestation; the children of women with the *VDR Apa*I SNP’s Aa genotype were born with a lower weight; women with *VDR Taq*I SNP’s *Tt* genotype decreased the risk of a shorter gestation duration; while women with *VDR Apa*I SNP’s aa genotype were negatively affected and had decreased gestation duration. Another case-control study (101 T2DM patients and 62 sex-, age-, and BMI- matched non-diabetic controls) from Brazil’s southeastern region evaluated the association between the *VDR* gene’s *Bsm*I, *Apa*I, *Fok*I, and *Taq*I polymorphisms in T2M patients and 25(OH)D serum levels. They suggested that Brazilian T2DM patients presented lower 25(OH)D serum levels unrelated to obesity and *VDR* polymorphisms [94].

Nevertheless, VDR expression and its role in transactivating target genes is determined not only by genetics but also by ethnicity and environment involving complex interactions which may confound disease association. O′Neill et al. (2013) [85] hypothesized that VDR expression/level and its target genes’ (*CAMP* and *CYP24A1*) transactivation hinge on the *Fok*I genotype, ethnicity, and vitamin D levels and found that differential *VDR* expression relates to ethnicity rather than 25(OH)D3 serum level and *Fok*I genotype as well as that *Fok*I polymorphism influenced *CAMP*’s VDR transactivation and, together with ethnicity, affects 1,25(OH)2D3-elicited *CYP24A1* induction.

For instance, the *Fok*I polymorphism also correlated with differences in TBBMD, but whereas some papers linked the more extended protein form presence with lower TBBMD [95,96], others liked the shorter form [97,98].

This study considered a sample of older women who underwent bariatric surgery and looked at the changes in vitamin D levels in these patients. However, a series of new questions have arisen that researchers should investigate: Are these hormone levels maintained after other years? Are the possible biological alterations due to this intervention, in terms of vitamin D metabolism, more impactful in this age group compared to the others?

## 5. Conclusions

In older Brazilian women who underwent bariatric Roux-en-Y gastric bypass surgery, after twelve months, 25(OH)D3 serum levels were positively correlated with total body bone mineral density, negatively with systolic blood pressure measurement, and their levels’ production was higher in those with the VDR *Fok*I polymorphism’s C (*f*) allele.

## Figures and Tables

**Table 1:** Correlation between vitamin D [25(OH)D] serum levels and biochemical, immunological, and anthropometric parameters.

25(OH)D (ng/mL) x	ρ	P	Parameters
Magnesium (mg/dL)	−0.182	0.516	Biochemical parameters
Vitamin B12 (pg/mL)	0.125	0.657	
TSH (mUI/L)	−0.057	0.84	
T3 (ng/dL)	−0.008	0.977	
T4 total (µg/dL)	−0.097	0.732	
Insulin (mU/L)	0.057	0.841	
Fasting blood glucose (mg/dL)	0.081	0.776	
Total cholesterol (mg/dL)	−0.171	0.545	
Triglycerides (mg/dL)	−0.397	0.143	
HDL (mg/dL)	−0.297	0.283	
LDL (mg/dL)	0.068	0.811	
VLDL (mg/dL)	−0.397	0.143	
Non-HDL cholesterol (mg/dL)	−0.054	0.849	
Total lipids (mg/dL)	−0.296	0.283	
Uric acid (mg/dL)	−0.106	0.707	
Sodium (mEq/L)	0.089	0.751	
Potassium (mEq/L)	0.102	0.718	
Chlorine (mEq/L)	−0.068	0.811	
Calcium (mg/dL)	0.368	0.177	
IL-2 (pg/mL)	0.189	0.499	Immunological parameters
TNF-α (pg/mL)	−0.051	0.861	
IL-6 (pg/mL)	0.368	0.177	
IL-10 (pg/mL)	0.404	0.136	
Fat mass (FM, g)	0.343	0.211	Anthropometric parameters
Lean body mass (LBM, g)	0.136	0.631	
Total body bone mineral density (TBBMD, g)	0.514	0.049 *	
Systolic pressure (mmHg)	−0.711	0.010 *	
Diastolic pressure (mmHg)	−0.311	0.327	
Body mass index (BMI, kg/m^−2^)	−0.051	0.861	

* P < 0.05—Spearman correlation coefficient; ρ: Spearman’s rank correlation coefficient; TSH = Thyroid-stimulating hormone; T3 = Triiodothyronine; T4 = Thyroxine; HDL = High-density lipoprotein; LDL = Low-density lipoprotein; VLDL = Very low-density lipoprotein; IL-2 = interleukin 2; TNF-α = Tumor necrosis factor alpha; IL-6 = interleukin 6; IL-10 = interleukin 10.

**Table 2:** Participants’ vitamin D [25(OH)D] serum levels according to the presence/absence of other clinical characteristics, symptoms, or habits.

Clinical Characteristics, Symptoms, or Habits		25(OH)D (ng/mL)				
		P25	Median	P75	N	P
Smoker	yes	20.20	27.10	34.00	2	
	no	20.65	26.70	35.15	18	0.983
	ex-smoker	25.20	26.30	27.00	7	
Alcoholic	yes	23.70	28.85	34.00	2	0.999
	no	22.30	26.30	30.60	25	
Hypertension	yes	22.30	26.25	34.00	23	0.933
	no	27.00	27.00	27.00	4	
Depression/anxiety	yes	20.20	26.65	30.60	11	0.864
	no	23.70	26.20	34.00	16	
Fibromyalgia	yes	22.30	39.70	40.70	4	0.233
	no	21.95	26.25	28.90	23	
Dyslipidemia	yes	19.00	21.35	23.70	5	0.171
	no	25.20	27.00	34.00	22	
Vaginal dryness	yes	24.80	32.80	37.35	6	0.343
	no	22.30	26.20	27.20	21	
Retinopathy	yes	22.30	26.20	27.20	6	0.734
	no	21.95	26.65	34.50	21	
Nephropathy	yes	--	--	--	0	NA
	no	22.30	26.30	34.00	27	
Neuropathy	yes	20.20	20.20	20.20	3	0.401
	no	23.70	26.65	34.00	24	
Arthralgia or myalgia	yes	22.30	27.00	35.00	10	0.779
	no	21.95	26.25	30.60	17	
Dysphagia or dyspepsia	yes	25.20	32.45	39.70	3	0.571
	no	22.30	26.30	30.60	24	
Frequent thirst	yes	23.70	26.20	30.60	11	0.867
	no	21.25	26.75	34.50	16	
Difficulty chewing dry food	yes	27.00	30.60	39.70	8	0.206
	no	22.30	25.70	27.20	19	
Difficulty speaking	yes	25.20	27.20	30.60	5	0.734
	no	21.25	26.25	34.50	22	
Gets up at night to drink water	yes	23.70	26.65	35.00	13	0.513
	no	20.20	26.20	30.60	14	
Loss or alteration of taste	yes	25.20	27.10	34.00	10	0.607
	no	22.30	26.20	30.60	17	
Xerostomia (dry mouth)	yes	25.20	26.30	34.00	13	0.607
	no	20.20	24.65	30.60	14	
Dry eyes	yes	23.70	26.30	34.00	13	0.776
	no	20.20	26.60	30.60	14	
Eye irritations	yes	23.70	26.30	34.00	12	0.779
	no	19.60	26.60	32.80	15	
Photophobia	yes	21.95	25.70	30.50	15	0.463
	no	22.30	27.20	39.70	12	
Myopia/hyperopia	no	20.20	27.10	34.00	4	0.999
	yes	23.70	26.30	30.60	23	
Eye drops	yes	23.00	25.00	26.75	5	0.571
	no	20.20	27.00	35.00	22	
Skin dryness	yes	25.20	27.20	35.00	17	0.145
	no	19.00	24.95	26.30	10	
Itch or rash (pruritus)	yes	22.70	27.90	32.30	6	0.999
	no	22.30	26.30	35.00	21	
Cracks (fissures) or red spots	yes	20.20	25.20	39.70	4	0.945
	no	23.00	26.65	32.30	23	
Tingling or numbness	yes	23.70	26.25	30.60	17	0.859
	no	20.20	27.00	34.00	10	
Lower limb pain	yes	23.70	26.30	35.00	19	0.851
	no	21.25	26.45	32.30	8	
Decrease perspiration	yes	22.30	23.70	30.60	4	0.633
	no	22.70	26.65	34.50	23	
Altered sexual performance	yes	19.00	22.30	27.20	8	0.295
	no	24.45	26.65	34.50	19	

Note: P25: 25th percentile; P75: 75th percentile.

**Table 3:** *VDR Taq*I and *VDR Fok*I polymorphisms’ genotypic distribution, according to the participants’ vitamin D [25(OH)D] serum level distribution and total body bone mineral density (TBBMD).

*VDR* Polymorphism				25(OH)D (ng/mL)				Total Body Bone Mineral Density (TBBMD, g)			
		N	P (HW)	P25	Median	P75	P	P25	Median	P75	P
*Taq*I	*TT*	12		22.30	27.20	35.00		1926.5	2112.5	2388.0	
	*Tt*	13	0.266	12.50	26.20	27.00	0.592	1903.0	1990.0	2336.0	0.835
	*tt*	2		26.30	26.30	26.30		1757.0	2064.5	2372.0	
	*TT*	12	NA	22.30	27.20	35.00	0.412	1926.5	2112.5	2388.0	0.581
	*Tt + tt*	15		19.35	26.25	26.65		1849.0	1990.0	2372.0	
*Fok*I	*FF*	10		15.75a	19.60	21.25		1733.0	2141.0	2501.0	
	*Ff*	15	0.257	26.20b	27.00	30.60	0.005 *	1903.0	1953.0	2336.0	0.217
	*ff*	2		39.70c	40.20	40.70		2370.0	2437.5	2505.0	
	*FF*	10	NA	15.75a	19.60	21.25	0.001 ^#^	1733.0	2141.0	2501.0	0.711
	*Ff+ff*	17		26.20b	27.20	35.00		1913.0	1973.0	2370.0	

Note: P25—25th percentile; P75—75th percentile; HW—Hardy-Weinberg equilibrium. Different letters denote statistical differences. * P < 0.005, Kruskal-Wallis H Test. ^#^ P < 0.005, Mann-Whitney U test.

## Data Availability

The research data are contained in the article’s tables.
